# Destruction of the Membrana Tympani and Application of the Toynbee Disc

**Published:** 1881-06-20

**Authors:** A. S. Core

**Affiliations:** Quincy, Ill.


### DESTRUCTION OF THE MEMBRANA TYMPANI, AND APPLICATION OF THE TOYNBEE DISC.

BY A. S. CORE, M. D., QUINCY, ILL.

*Case No.* 10.—Age 24. Came in for treatment, complaining of a discharge from both ears; stating that the discharge had existed since at about one year of age, and that it could not be stopped; had been informed that if the discharge was stopped death would be the result. I see that the patient is fairly nourished. State of hearing for a watch, 1-60 in the right ear, and 1-60 in the left ear. The tuning fork is heard best when placed on the incisor teeth or vertex. On examination of the middle ear with the Otoscope, I find that the membrana tympani is entirely destroyed; the ossicles intact at the foramen ovale, but greatly drawn down from their natural positions, and adhesions extending from the point of the handle of the malleus to the base of the bone, drawing it down firmly and closely to the posterior wall of the middle ear; this condition existing in both ears, with a very fetid discharge of pus.

With the rhinoscope, could be seen, in the post-pharyngeal space and at the opening of the eustachian tubes, a granulated surface, with dry crusts on some of the eminences of the parts. Nasal passages, anteriorly, were normal; throat and larynx normal. I attempted to inflate the eustachian tubes, both by Politzer's method and the eustachian catheter, but could not. I then made direct application to the parts of the post-pharyngeal space, with the brush, of a solution containing two per cent. of the chloride of zinc; syringed the ears well, and applied a weak solution of permanganate of potassium. In about one week I was enabled to inflate both the eustachian tubes, and in about one month all discharge had stopped, and the parts began to assume a healthy appearance; hearing had been improved so that the patient could hear the watch at the distance of 12-60 in both ears, with considerable improvement in conversation. Before the eustachian tubes were opened, all tones on the lower register were heard with an autophonous sound; after the tubes had been opened, and retained so, the autophony ceased; but tones on the lower register were not appreciated quite as soon as those of the middle or upper register, but always normal. The patient at no time complained of any unpleasant sensation caused by hearing her own footsteps on the pavement, or the heavy rumbling sounds of the street. I now applied the Toynbee discs in both ears, and after some manipulation of the discs, so as to have them set easy and cause no pain, I found that there was a point somewhere on the malleus or its attachments, of the right ear, that when the disc was placed right it would increase the hearing, so the patient could hear the watch at a distance of 30-60. I tried the left ear and found that the same result could be attained in that ear. The next day, when the patient had become accustomed to the discs, the watch could be heard at a distance of 45-60, and the patient could converse in ordinary conversation, common tones, as well as any one. I now informed the patient that it would not be necessary to call so often, and gave instruction to take the discs out at night, and clean them, and introduce them again in the morning, until the ears had become well accustomed to their presence. This advice was disregarded, the patient being so well pleased with the result thought it would not be necessary to change the discs so often, and wore them for ten days or two weeks without any change; as a result, they caused an irritation, and re-established the discharge. After the discharge was again stopped, or partially so, I found that cotton-wool pellets, saturated with glycerine, would answer almost the same purpose as that of the discs; but they were harder to adjust, and would not retain the moisture and their place so well.

The patient, when last seen, had no discharge from the ears, and could hear very well when using the discs; the hearing for the watch, without the discs, was the same as when the discharge was first stopped.

The Toynbee disc is the *sine qua non* for all aural catarrh. I regard its greatest use when there is entire or almost complete destruction of the membrana tympani, and the ossicles still remaining. In perforations, falling, acute suppuration, with no great destruction of the membrana tympani, I prefer to use simply a disc, cut from sizing or parchment paper, slightly moistened, and placed over the perforation. I have several times seen perforations heal under the use of the paper discs, with no other treatment than gentle syringing with warm water.

About the introduction of the discs (see Toynbee's text book), except after it is in position I often find it advantageous to apply the air-bag to the external meatus, and by a quick blow drive the disc against the remains of the middle ear. The full improvement of hearing is rarely attained at once, it may be half an hour, perhaps longer, after the introduction of the disc, before any change is observed. After the surgeon has once trimmed and fitted the disc, the patients, if intelligent, need experience no inconvenience in introducing them themselves. My observations have been that the greatest improvement in hearing has more often been a benefit for the voice than for the watch.—*Med. Call.*

